# Enhanced Inversion for Distributed Acoustic Sensing: A Robust Approach with HOLp–OGS Regularization

**DOI:** 10.3390/s26134051

**Published:** 2026-06-25

**Authors:** Wenhua Xu, Jingye Li, Yaning Wu, Weiheng Geng, Bangbang Gao, Lei Han

**Affiliations:** 1 State Key Laboratory of Petroleum Resources and Prospecting, College of Geophysics, China University of Petroleum (Beijing), Beijing 102249, China; 2021310417@student.cup.edu.cn (W.X.); 18717315155@163.com (B.G.); 2023315403@student.cup.edu.cn (L.H.); 2 CNOOC China Limited, Tianjin Branch, Tianjin 100083, China; wuyn4@cnooc.com.cn; 3 College of Artificial Intelligence, China University of Petroleum (Beijing), Beijing 102249, China; gengwh@cup.edu.cn

**Keywords:** DAS-to-geophone conversion, distributed acoustic sensing, high-order Lp quasi-norm, overlapping group sparsity regularization

## Abstract

Conversion from distributed acoustic sensing (DAS) measurements to geophone-equivalent data is important for integrating DAS into conventional seismic workflows. This is because most established seismic-processing algorithms are designed for particle-velocity or acceleration data, whereas DAS measures strain or strain rate. Recovering geophone-equivalent particle velocity from DAS strain-rate measurements requires inversion of a gauge-length-dependent spatial-difference operator, which can amplify measurement noise, particularly in field data with low signal-to-noise ratios (SNRs). Existing single-regularization methods often trade noise attenuation against waveform fidelity and the preservation of weak coherent events. To address these limitations, we propose an inverse reconstruction framework combining high-order *L_p_* (HOLp) and overlapping group sparsity (OGS) regularizations. HOLp promotes a compact representation of second-order differences and suppresses incoherent fluctuations, whereas OGS exploits local coherence to reduce isolated artifacts and preserve weak continuous events. The resulting objective function is solved using the alternating direction method of multipliers, with iteratively reweighted *L*_1_ minimization for the HOLp subproblem and a majorization–minimization strategy for the OGS subproblem. Numerical and field experiments confirm that the method restores amplitude and waveform fidelity under low SNR conditions, demonstrating robust and reliable DAS-to-geophone conversion.

## 1. Introduction

Distributed acoustic sensing (DAS) has become an important seismic sensing technology across a broad range of geophysical and engineering applications, including seismic exploration, microseismic monitoring, reservoir surveillance, geothermal and CO_2_ storage monitoring, earthquake studies, and near-surface or infrastructure monitoring [1,2,3]. Unlike conventional geophones, which measure particle velocity or acceleration, DAS systems measure strain or strain rate through optical phase shifts [4,5]. This fundamental discrepancy necessitates DAS-to-geophone conversion to ensure compatibility with conventional velocity-based seismic workflows, including processing and migration [6,7,8].

DAS measurements inherently exhibit gauge-length effects [9], inducing spatial averaging over finite fiber segments [10]. This spatial averaging acts as a low-pass filter, attenuating high-frequency components and degrading waveform fidelity [10,11]. Some studies suggest that the gauge-length effect should be incorporated into the conversion process to achieve high-precision conversion results [7]. Moreover, DAS-acquired data typically exhibit lower signal-to-noise ratios (SNRs) than geophone recordings and suffer from unique noise sources (e.g., optical fading), limiting conventional denoising efficacy [12,13,14]. Consequently, these effects degrade DAS-to-geophone conversion fidelity, necessitating robust conversion methods. When the gauge-length effect is compensated for, these noise components may be amplified because the conversion problem is ill-conditioned. Robust regularization is therefore needed to stabilize the reconstruction of geophone-equivalent particle-velocity data from DAS strain-rate measurements.

Several methods have been developed for DAS-to-geophone data conversion, including local phase velocity estimation [15], slant-stacking-based slowness analysis [16], spatial integration [17,18], f-k domain techniques [19,20], and deep-learning-based transformations [21]. These methods can be effective under favorable conditions. However, their performance often degrades for noisy field data or complex wavefields. Spatial integration can produce relatively consistent results, but it may accumulate low-frequency drift and interfering noise, which usually requires additional filtering. F-k domain methods rely on sufficient spectral separation between useful events and noise. Their performance may therefore degrade when weak reflections, steeply dipping events, and noise overlap in the frequency-wavenumber domain. Deep learning-based methods provide another possible solution, but their performance depends on high-quality labels and transferability across survey areas. These limitations motivate a physics-guided inverse reconstruction framework that explicitly accounts for the DAS measurement response and stabilizes the recovery through appropriate prior constraints. Throughout this paper, inverse reconstruction denotes the regularized data-domain estimation of geophone-equivalent particle-velocity traces from DAS strain-rate measurements [22], rather than conventional seismic inversion for subsurface velocity, impedance, or other physical-property models.

Monsegny et al. [22] developed inversion-based DAS-to-geophone conversion methods using *L*_2_-norm and first-order Tikhonov regularization. By incorporating the finite-gauge-length DAS response into the forward operator, these methods provide a physically consistent basis for reconstructing geophone-equivalent particle velocity. Regularization stabilizes the reconstruction and limits noise amplification but also introduces prior-dependent bias. Regularization stabilizes the reconstruction by limiting noise-driven solutions, but it also introduces constraint-dependent bias. The *L*_2_ penalty favors a minimum-energy solution and may attenuate signal amplitudes, whereas first-order Tikhonov regularization favors a smooth solution and may smear sharp arrivals or weak events [23]. Consequently, either regularization may produce a trade-off between noise attenuation and waveform preservation, motivating the use of complementary structural constraints.

Sparsity-promoting regularization provides an alternative means of stabilizing the reconstruction. The *L*_0_-regularized optimization is nonconvex and combinatorial, which makes it impractical for large-scale DAS applications [24,25]. The *L*_1_ norm is a widely used convex surrogate for the *L*_0_ quasi-norm [26]. Although computationally tractable, it may provide suboptimal sparsity by retaining many small nonzero coefficients in the reconstructed wavefield and does not explicitly exploit local wavefield structure [27]. This may lead to amplitude bias or smeared wavefield features, especially when weak coherent events coexist with noise. By contrast, nonconvex *L_p_*-type penalties with 0 < *p* < 1 are closer to the *L*_0_ quasi-norm and can promote more compact representations [27]. They have been used in geophysical inverse problems to improve sparsity and noise attenuation [28,29,30].

To balance noise suppression and waveform preservation, we propose a DAS-to-geophone data conversion method with HOLp–OGS regularization. The proposed regularization combines higher-order *L_p_* (HOLp) sparsity with overlapping group sparsity (OGS), imposing complementary constraints on compact representation and local structural coherence. The HOLp term promotes stronger sparsity than conventional sparsity penalties, thereby suppressing incoherent fluctuations during data conversion. However, excessive sparsity may cause amplitude instability, attenuation of weak events, or isolated artifacts because it does not explicitly account for the spatial coherence of seismic wavefields. To address this limitation, OGS regularization is introduced to constrain neighboring samples through overlapping groups and exploit the spatial continuity of seismic events. It has found extensive application across various fields, including compressed sensing [31], machine learning [32], seismic inversion [33,34], image reconstruction [35,36], signal denoising [37,38], and ground-penetrating radar detection [39]. By exploiting local group structures, OGS helps preserve weak but coherent wavefield components and suppress isolated artifacts. Therefore, the combined HOLp–OGS regularization improves noise robustness while maintaining waveform fidelity and structural continuity.

The inversion problem is formulated within the alternating direction method of multipliers (ADMM) framework to enable efficient decoupling of constraints and stable convergence. The HOLp term is solved via the iteratively reweighted *L*_1_ (IRL_1_) scheme, while the OGS term is handled using a majorization–minimization (MM) strategy. Synthetic tests and field data both confirm that the method yields high-fidelity DAS-to-geophone conversion, effectively compensating for gauge-length distortion, recovering amplitude, and correcting waveform polarity. These results highlight the potential of the proposed method for accurate and robust DAS-to-geophone conversion in DAS-based seismic workflows.

## 2. Theory and Methods

### 2.1. DAS Theory and Conversion

The forward modeling principle for DAS is commonly described by the following equation, which follows the formulation proposed by Hartog [40]:

(1) $${\dot{\varepsilon }}_{z}(i,t)=\frac{v_{z}\left(i+\frac{g}{2},t\right)-v_{z}(i-\frac{g}{2},t)}{g},$$

where ${\dot{\varepsilon }}_{z}$ denotes the DAS-acquired strain-rate data, *v_z_* denotes velocity, *z* represents fiber’s axial direction, *g* is the gauge length, *t* denotes time, and *i* refers to a specific sampling point along the fiber. Evidently, the measurement at point *i* represents the average strain rate over the gauge length centered at that location, rather than the exact strain-rate value at *i*. The approach of substituting exact values with averages will alter the waveform of the measured data and cause the amplitude values to deviate from the original ones. This effect is referred to as the gauge-length effect. To achieve more accurate conversion results, this effect must be accounted for and addressed during the conversion process.

Applying the discretized finite-gauge-length observation relation to each time sample and arranging the traces as matrices give Equation (2):

(2) d=GS+n.

where **d** denotes the DAS strain-rate data, **S** denotes geophone-equivalent particle-velocity data to be reconstructed, **G** is the gauge-length-dependent forward operator, and **n** represents additive measurement noise. Here, inversion refers to regularized data-domain reconstruction rather than conventional seismic inversion for subsurface physical-property estimation [22]. A similar formulation can also be established for strain-to-displacement conversion, whereas this study focuses on DAS strain-rate-to-geophone-equivalent particle-velocity conversion. Therefore, Equation (2) is the discretized and stacked form of the finite-gauge-length DAS response in Equation (1) and is consistent with the least-squares DAS-to-geophone transform concept in [22]. The operator **G** is defined as

(3) $$G=\frac{1}{g}\left[\begin{array}{ccccccc} -1 & 0 & \cdots & 1 & 0 & \cdots & 0\\ 0 & -1 & 0 & \cdots & 1 & \cdots & 0\\ \vdots & & & \ddots & & & \vdots \\ 0 & \cdots & 0 & -1 & 0 & \cdots & 1 \end{array}\right].$$

Although Equation (2) is linear, directly recovering **S** from **d** is ill-conditioned. Regularization is therefore introduced to stabilize the reconstruction of geophone-equivalent particle-velocity data.

### 2.2. Inversion Objective Function Construction

From a Bayesian perspective, the inverse reconstruction can be formulated by combining a likelihood function with prior constraints on the unknown geophone-equivalent particle-velocity data, **S**. According to Bayes’ theorem, the posterior probability distribution of S given the observed DAS data **d** is [41,42,43,44]

(4) $$P\left(S|d\right)=\frac{P\left(d|S\right)P\left(S\right)}{P\left(d\right)}.$$

Here, $P\left(d|S\right)$ is the likelihood function, $P\left(S\right)$ is the prior distribution, and $P\left(d\right)$ is the evidence term independent of **S**. For the observation equation d=GS+n, **n** is modeled as zero-mean Gaussian noise [45,46,47]. Therefore, the likelihood function can be written as

(5) $$P(d|S)\propto \exp\left[-\frac{1}{2\sigma_{n}^{2}}{\left\|GS-d\right\|}_{2}^{2}\right],$$

**Figure 1:**
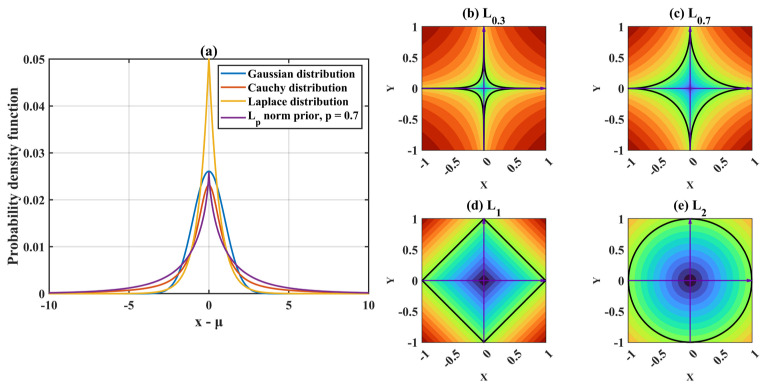
Comparison of probability density functions and norm-contour geometries. (**a**) Probability density functions of Gaussian, Cauchy, Laplace, and *L_p_*-norm priors. (**b**–**e**) Contour illustrations of the *L*_0.3_, *L*_0.7_, *L*_1_, and *L*_2_ norms, respectively.

This likelihood leads to the *L_2_* data-misfit term. The HOLp and OGS regularizations introduced below are interpreted as structural prior constraints on **S**, whereas the data-dependent reference term is introduced separately as a reference constraint. A general prior on **S** can be expressed, up to a normalization constant, as

(6) $$P\left(S\right)\propto \exp\left[-R\left(S\right)\right],$$

where $R\left(S\right)$ is a regularization function imposed on **S**. Different choices of prior distributions correspond to different forms of $R\left(S\right)$. As illustrated in Figure 1a, Gaussian and Laplace priors correspond to *L*_2_- and *L*_1_-type penalties, respectively, whereas the *L_p_*-type prior with 0 < *p* < 1 is more sharply concentrated near zero and exhibits heavier tails, thereby providing stronger sparsity promotion while imposing less shrinkage on significant coefficients. In this study, the total regularization consists of the prior-derived HOLp and OGS penalties and a data-adaptive reference-model constraint. Combining Bayes’ theorem, the Gaussian likelihood, and the prior gives the posterior distribution associated with the data-misfit and regularization terms.

(7) $$P\left(S|d\right)\propto \exp[-\frac{1}{2\sigma_{n}^{2}}\left(GS-d{)}^{T}\left(GS-d\right)-R\left(S\right)\right]$$

where $\sigma_{n}$ denotes the noise standard deviation. In Equation (7), the evidence term $P\left(d\right)$ is omitted because it is independent of **S**. The solution that maximizes the posterior probability is the maximum a posteriori estimate. After taking the negative logarithm of the Gaussian working likelihood and applying an equivalent rescaling, the resulting penalized objective is expressed as

(8) $$J\left(S\right)=\underset{S}{\min}\;{\left\|GS-d\right\|}_{2}^{2}+R\left(S\right).$$

To address the non-uniqueness and mitigate the instability of the inverse problem, we introduce an integrated regularization framework that combines the HOLp pseudo-norm with OGS. For a given seismic signal **S**, the *L_p_* pseudo-norm is defined as [48]

(9) $${\left\|S\right\|}_{p}^{p}=\sum_{i=1}^{N}\sum_{j=1}^{N}{\left|S_{ij}\right|}^{p},\;0<p<1.$$

**Figure 2:**
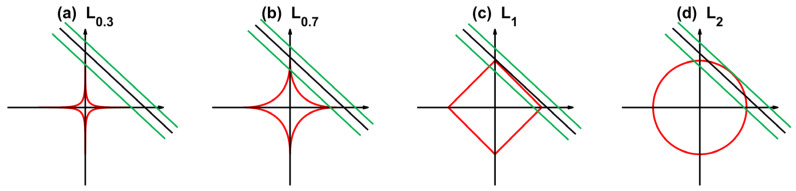
Feasible regions of different norms: (**a**) *L*_0.3_, (**b**) *L*_0.7_, (**c**) *L*_1_, (**d**) *L*_2_.

The superiority of the *L_p_* pseudo-norm over conventional norms is demonstrated in Figure 1b–e and Figure 2, which depict schematic contour plots and feasible regions of the objective functions. Figure 1a,b correspond to *L_p_* norms with *p* = 0.3 and 0.7, whereas Figure 1c,d illustrate the *L*_1_ and *L*_2_ norms. In Figure 2, the black line denotes the data fidelity constraint. The *L*_1_ norm produces diamond-shaped contours, and the *L*_2_ norm producescircular ones, while the *L_p_* norm yields sharper, nonconvex geometries that more effectively promote sparsity—an effect that intensifies as *p* decreases.

**Figure 3:**
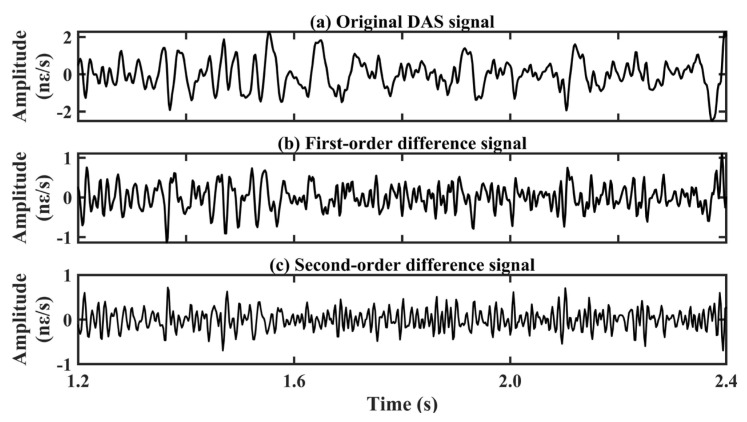
Sparsity comparison using the field DAS strain-rate data (sampling interval: 2 ms; sampling frequency: 500 Hz; duration: 4 s). The first- and second-order discrete differences were computed with a one-sample difference step. (**a**) Original DAS strain-rate trace; (**b**) first-order discrete difference; and (**c**) second-order discrete difference.

When seismic data is collected using geophones or DAS, the continuous smooth waveform is influenced by the time sampling interval. Although it sometimes exhibits a piecewise linear characteristic, it remains a generally smooth continuous waveform with relatively poor sparsity. To illustrate the sparsity behavior of DAS data and their difference-domain representations, we use a field DAS strain-rate trace rather than a synthetic signal. The trace has a sampling interval of 2 ms, a sampling frequency of 500 Hz, and a 4 s recording window. As shown in Figure 3, the first- and second-order temporal-difference traces were computed from neighboring samples using the corresponding discrete-difference matrices with a one-sample difference step. Using the sparsity evaluation formula in Equation (10), proposed by Hurley and Rickard, the sparsity of the three signals was quantitatively evaluated [49]. The sparsity values are found to be 25.7 (raw signal), 19.9 (first-order difference signal), and 15.1 (second-order difference signal), respectively, indicating that the second-order difference signal, which exhibits a piecewise linear characteristic, has stronger sparsity.

(10) $$H_{1,2}=\frac{l_{1}\left(d\right)}{l_{2}\left(d\right)}.$$

where $H_{1,2}$ represents the sparsity measure, ***d*** is the signal, $l_{1}\left(d\right)$ is the *L*_1_ norm, and $l_{2}\left(d\right)$ is the *L*_2_ norm. To exploit this property, we introduce the following HOLp pseudo-norm regularization term by combining the second derivative of the data with the *L_p_* pseudo-norm:

(11) $$\phi (S)={\left\|G_{s}S\right\|}_{p}^{p},$$

where **G**_s_ is the second-order difference operator, defined as follows:

(12) $$G_{s}=\left[\begin{array}{llllll} -1 & 2 & -1 & \cdots & 0 & 0\\ 0 & -1 & 2 & -1 & \cdots & 0\\ 0 & 0 & -1 & 2 & \cdots & 0\\ \vdots & \ddots & \ddots & \ddots & \ddots & \vdots \\ 0 & 0 & 0 & \cdots & -1 & 2\\ 0 & 0 & 0 & 0 & \cdots & -1 \end{array}\right],$$

The HOLp regularization term defined in Equation (11) imposes a heavy-tailed sparsity assumption on the second-order difference coefficients:

(13) $$P_{\phi }\left(S\right)\propto \exp\left[-\mu \phi \left(S\right)\right].$$

Compared with *L*_1_ regularization, the nonconvex *L_p_* penalty promotes a more compact representation and reduces excessive shrinkage of large coefficients. It therefore helps suppress small incoherent fluctuations while retaining pronounced waveform variations.

In addition to the HOLp constraints, we introduce a weak data-domain reference term. **S**_ref_ is not a subsurface velocity or impedance model; it is a smooth reference estimate of the geophone-equivalent particle-velocity data. Depending on data availability, **S**_ref_ can be constructed either from a stable preliminary DAS-to-geophone conversion, followed by specified smoothing, or from interpolation of independent co-located geophone measurements. In this study, we use the former. The reference contains only broad waveform trends and is not intended to impose detailed reflection structures. Its role is to weakly guide the reconstruction and reduce unstable deviations from a physically plausible reference. Because **S**_ref_ is constructed by smoothing a preliminary conversion of the same DAS observations, the quadratic reference term is treated as a data-adaptive reference constraint rather than a data-independent prior.

(14) $$P_{\mathrm{ref}}\left(S\left|S_{\mathrm{ref}}\left(d\right)\right.\right)\propto \exp\left[-\delta ∥S-S_{\mathrm{ref}}\left(d\right){∥}_{2}^{2}\right].$$

Combining the *L*_2_ data-misfit term, the quadratic reference term, and the HOLp penalty yields the following objective:

(15) $$J\left(S\right)=\underset{S}{\min}\;{\left\|\mathrm{GS}-d\right\|}_{2}^{2}+\delta {\left\|S-S_{\mathrm{ref}}\right\|}_{2}^{2}+\mu \phi \left(S\right).$$

where **d** denotes the observed field data and **S**_ref_ denotes the smooth data-domain reference constructed using the procedure described above. The parameter *δ* is a regularization weight controlling the strength of the reference constraint. The reference term weakly guides the reconstruction toward a stable reference estimate and does not represent a subsurface velocity or impedance model.

Although the first-order derivative of seismic signals is globally less sparse than the second-order derivative, it exhibits pronounced local sparsity and structured behavior. In particular, sparse features in the first-order derivative tend to cluster, forming a characteristic group-sparse pattern consistent with the group sparsity theory of Ivan and Chen. To exploit this prior, we incorporate OGS into the DAS-to-geophone conversion inversion framework. This integration leverages neighborhood information to improve the reconstruction of spatially coherent seismic features. The OGS structure is defined as follows [33,34]:

(16) $$\mathrm{OGS}(U)=\left(\sum_{j=0}^{J-1}|U(1+j){|}^{2},\sum_{j=0}^{J-1}|U(2+j){|}^{2},\cdots ,\sum_{j=0}^{J-1}|U(m+j){|}^{2}\right),m=1,2,\ldots ,M,$$

where $U\left(m+j\right)=DS\left(m+j\right)$ and *m* denotes the step size of the signal groups (i.e., the length of overlap between adjacent groups), which is typically set to 1 for computational simplicity. *J* represents the length of each group, and *M* denotes the total length of the signal. **D** is the first-order difference operator, defined as follows:

(17) $$D={\left[\begin{array}{ccccc} -1 & 1 & 0 & \cdots & 0\\ \vdots & -1 & 1 & \ddots & \vdots \\ \vdots & \ddots & \ddots & \ddots & 0\\ 0 & \cdots & \cdots & -1 & 1 \end{array}\right]}_{\left(M-1\right)\times M},$$

**Figure 4:**
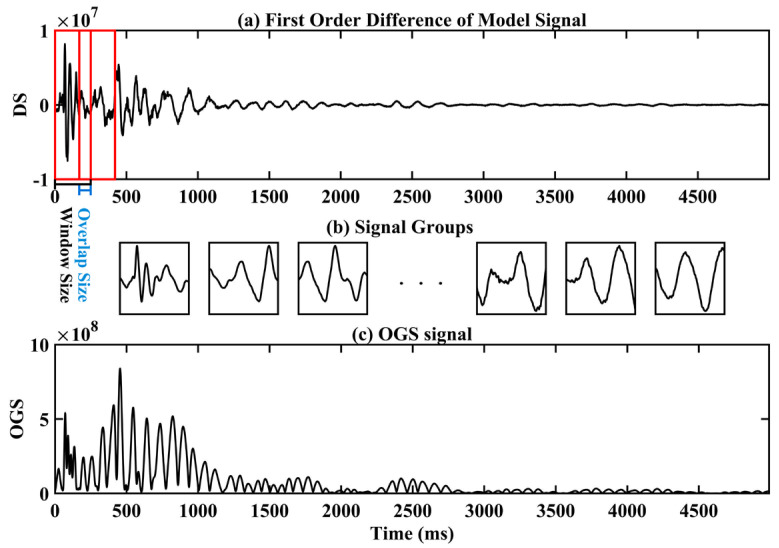
Schematic diagram of OGS. (**a**) First-order difference of the time-domain model DAS signal; (**b**) signal segments grouped from (**a**) via a sliding window with group size *J*; (**c**) OGS signal derived from (**b**) using Equation (16).

Figure 4 illustrates the principle of OGS. The processing of a DAS signal begins with computing its first-order difference signal (Figure 4a). This differenced signal is then segmented into multiple signal groups using a sliding window with step size *J*. Adjacent windows overlap during the grouping process, generating overlapping sparse groups that enhance the continuity of the group-sparse signal and effectively capture local features and structural information (Figure 4b). Finally, the overlapping group-sparse signal is obtained by applying the group-sparsity constraint defined in Equation (16) to each signal group (Figure 4c).

The OGS regularization term $\varphi \left(S\right)$ can be associated with the following unnormalized prior form:

(18) $$P_{\varphi }\left(S\right)\propto \exp\left[-\lambda \varphi \left(S\right)\right].$$

This prior is imposed on overlapping groups of the first-order difference signal. It encourages neighboring samples to be jointly constrained and promotes local structural coherence. Therefore, it helps suppress isolated artifacts while preserving weak but spatially continuous seismic events. After introducing the OGS constraint into Equation (15), the final objective function can be expressed as follows:

(19) $$J_{\mathrm{HOLp-ogs}}\left(S\right)=\underset{S}{\min}\;{\left\|\mathrm{GS}-d\right\|}_{2}^{2}+\delta {\left\|S-S_{\mathrm{ref}}\right\|}_{2}^{2}+\mu \phi \left(S\right)+\lambda \varphi (S).$$

where φ(S) denotes the OGS regularization term and *λ* is its associated weighting parameter. The *L*_2_ data-misfit term is motivated by the Gaussian working likelihood, whereas the HOLp and OGS terms encode complementary structural prior assumptions. The quadratic reference term incorporates broad waveform information estimated from the same DAS observations. Consequently, the resulting estimator has an empirical-Bayes interpretation conditional on the estimated reference, but it should not be regarded as a strict MAP estimator based on a data-independent prior.

### 2.3. Proposed Objective Function Solution Method

The proposed objective contains multiple regularization terms that are conveniently handled by different numerical solvers. We therefore introduce auxiliary variables and equality constraints and optimize the resulting constrained formulation using ADMM. When the equality constraints are satisfied, the variable-splitting formulation is equivalent to the original objective and introduces no additional modeling error. With finite iterations, incomplete satisfaction of these constraints can produce a numerical splitting error, which is monitored using the constraint residuals and the relative change between successive reconstructions.

(20) $$\begin{array}{l} J_{\mathrm{HOLp-ogs}}(S)=\min{\left\|GS-d\right\|}_{2}^{2}+\delta {\left\|S-S_{ref}\right\|}_{2}^{2}+\mu \phi \left(W\right)+\lambda \varphi (R)\\ \mathrm{s.t.}\;W=G_{s}S,\;R=DS \end{array},$$

Within the ADMM framework, each subproblem in Equation (20) is solved by minimizing its associated objective function. The augmented Lagrangian formulation is expressed as:

(21) $$\begin{array}{cc} £\left(S,W,R;\alpha_{W},\alpha_{R}\right) & =\min{\left\|GS-d\right\|}_{2}^{2}+\delta {\left\|S-S_{ref}\right\|}_{2}^{2}\\ & +\mu \phi \left(W\right)-\beta \alpha_{W}^{T}\left(W-G_{s}S\right)+\frac{\beta }{2}{\left\|W-G_{s}S\right\|}_{2}^{2},\\ & +\lambda \varphi \left(R\right)-\beta \alpha_{R}^{T}\left(R-DS\right)+\frac{\beta }{2}{\left\|R-DS\right\|}_{2}^{2} \end{array}$$

where *β* is the penalty parameter that controls the influence of the penalty terms associated with the constraint variables ${\left\|W-G_{s}S\right\|}_{2}^{2}$ and ${\left\|R-DS\right\|}_{2}^{2}$. $\alpha_{W}$ and $\alpha_{R}$ are the Lagrange multipliers corresponding to the constraints defined in Equation (20). By further decomposing Equation (20) into a set of subproblems, we obtain the following formulations:

(22) $$\left\{\begin{array}{l} S^{n+1}=\underset{S}{\mathrm{argmin}\;}£\left(S,W^{n},R^{n};\alpha_{W}^{n},\alpha_{R}^{n}\right)\\ R^{n+1}=\underset{R}{\mathrm{argmin}\;}£\left(S^{n+1},W^{n},R;\alpha_{W}^{n},\alpha_{R}^{n}\right)\\ W^{n+1}=\underset{W}{\mathrm{argmin}\;}£\left(S^{n+1},W,R^{n+1};\alpha_{W}^{n},\alpha_{R}^{n}\right)\\ \alpha_{W}^{n+1}=\underset{\alpha_{W}}{\mathrm{argmin}\;}\beta \alpha_{W}^{T}\left(W-G_{s}S^{n+1}\right)\\ \alpha_{R}^{n+1}=\underset{\alpha_{R}}{\mathrm{argmin}\;}\beta \alpha_{R}^{T}\left(R-DS^{n+1}\right) \end{array}\right.,$$

where *n* denotes the iteration index of the ADMM update.

In this study, different algorithms are employed to solve the individual subproblems: specifically, the MM algorithm is used to solve subproblem **R** [37], while the IRL_1_ algorithm is applied to subproblem **W** [26]. Subproblem S follows a standard least-squares formulation. The respective iterative update formulations for the three subproblems are as follows:

(23) $$R^{n+1}={\left(\left(I+\frac{\beta }{\lambda })\Lambda {\left(R^{n}\right)}^{T}\Lambda (R^{n}\right)\right)}^{-1}(DS^{n}+\alpha_{R}^{n}),$$

(24) $$W^{n+1}=\mathrm{shrink}\;\left(D_{s}S^{n}+\alpha_{W}^{n},\frac{p{\left(\left|W^{n}\right|+10^{-7}\right)}^{p-1}\mu^{2}}{\beta }\right),$$

(25) $$\begin{array}{cc} S^{n+1} & ={\left(G^{T}G+\delta I+\beta {G_{s}}^{T}G_{s}+\beta D^{T}D\right)}^{-1}\\ & \;\left(\begin{array}{c} G^{T}d_{\mathrm{obs}}+\delta S_{\mathrm{ref}}+\\ \beta {G_{s}}^{T}\left(W^{n}-\alpha_{W}^{n}\right)+\beta D^{T}\left(R^{n}-\alpha_{R}^{n}\right) \end{array}\right), \end{array}$$

where the symbol Λ represents the calculation of the diagonal matrix, as detailed in the appendix, and shrink refers to the iterative soft-thresholding operation. The detailed solution process for Equations (23) and (24) can be found in Appendix A and Appendix B.

For the subproblems $\alpha_{W}$ and $\alpha_{R}$ in Equation (22), simple gradient descent methods suffice to derive the corresponding iterative update expressions [33], as follows:

(26) $$\begin{array}{l} \alpha_{W}^{n+1}=\alpha_{W}^{n}+\beta \left(W^{n+1}-G_{s}S^{n+1}\right)\\ \alpha_{R}^{n+1}=\alpha_{R}^{n}+\beta \left(R^{n+1}-DS^{n+1}\right). \end{array}$$

Given the large number of parameters involved, obtaining satisfactory inversion results often requires iterative tuning of the regularization weights. This process can be computationally intensive, as extensive testing is typically needed to identify optimal parameter values. Algorithm 1 outlines the pseudocode for solving the objective function. The ADMM penalty parameter *β* and stopping tolerance tol were selected empirically through preliminary tests and were then fixed.
**Algorithm 1:** HOLp-OGS Regularized Velocity-Strain Conversion**Input:**Observed DAS data **d_obs_**, Low-frequency model of DAS data **S_ref_**, OGS group size ***K***, Order of quasi-norm *p*, Convergence threshold *tol*, Regularization parameters (*λ*, *δ*, *μ*).**Intial:***n* = 0; *β* = 0.02; **S**^0^ = **S_ref_**; **R**^0^ = 0, **W**^0^ = 0; $\alpha_{W}^{0}=0;\alpha_{R}^{0}=0;\mathrm{tol}=10^{-6}$**Steps:**1:Compute the first-order and second-order difference matrix **D** and **G**_s_;2:While ${{\left\|S^{n+1}-S^{n}\right\|}_{2}/\left\|S^{n}\right\|}_{2}>\mathrm{tol},$do:3:Update **S**, **W**, and **R** by (16), (15), and (14), respectively;4:Update $\alpha_{W}^{n+1}$$\mathrm{and}\;\alpha_{R}^{n+1}$by (17);5:*n* = *n* + 1;6:end**Output:**Output the inversion seismic result S

## 3. Algorithm Testing and Validation

### 3.1. Tests for Parameter Selection

The objective function in the inversion process contains numerous hyperparameters, which are interrelated, making the widely used generalized cross-validation and L-curve methods impractical in seismic exploration. To select appropriate hyperparameters, trial-and-error methods, which offer high flexibility and adaptability, are typically employed. However, trial-and-error methods are not practical for large-scale DAS data. Therefore, a combination of the advantages of the L-curve method and trial-and-error methods can be used. In practice, trial-and-error methods are typically applied to test well-side seismic records to determine the optimal regularization parameters, which are then generalized across the entire survey area, thus improving computational efficiency while maintaining inversion accuracy.

Additionally, for a quantitative comparison, the correlation coefficient (CC), the root mean square error (RMSE) and SNR are used as evaluation metrics, defined as follows:

(27) $$CC=\frac{\sum_{i=1}^{nt}\left(S_{i}-\bar{S})({\hat{S}}_{i}-\bar{\hat{S}}\right)}{\sqrt{\sum_{i=1}^{nt}{\left(S_{i}-\bar{S}\right)}^{2}\sum_{i=1}^{nt}{\left({\hat{S}}_{i}-\bar{\hat{S}}\right)}^{2}}},$$

(28) $$SNR=10\times \mathrm{Log}\left(\frac{{\left\|d_{\mathrm{cle}}\right\|}_{2}^{2}}{{\left\|d_{\mathrm{noi}}-d_{\mathrm{cle}}\right\|}_{2}^{2}}\right),$$

(29) $$RMSE=\sqrt{\frac{1}{nt\times nx}\sum_{i=1}^{nt}\sum_{j=1}^{nx}{\left(S_{i,j}-{\hat{S}}_{i,j}\right)}^{2}}.$$

where $S_{i}$ and ${\hat{S}}_{i}$ denote the true model and inversion results at the *i*th sampling point, $\bar{\;\cdot \;}$ represents the averaging operator, *nt* is the number of time sampling points, **d_noi_** is the noisy DAS signal, and **d_cle_** is the clean DAS signal. *nx* is the number of seismic traces. $S_{i,j}$ and ${\hat{S}}_{i,j}$ represent the data at the *i*th time sample point of the *j*th trace for the true model and the inversion result, respectively.

To determine the optimal hyperparameters for the low-frequency background model term, the HOLp regularization term, and the OGS regularization term within the inversion objective function, a systematic parameter selection strategy was implemented. The primary focus was initially placed on the weight parameter of the low-frequency background model term to ensure the fidelity of its representation in the inversion results. For this purpose, trace number 350 from the synthetic model example—designated as the well tie trace—was selected for testing (the detailed generation process of the synthetic model will be elaborated in Section 3.2).

**Figure 5:**
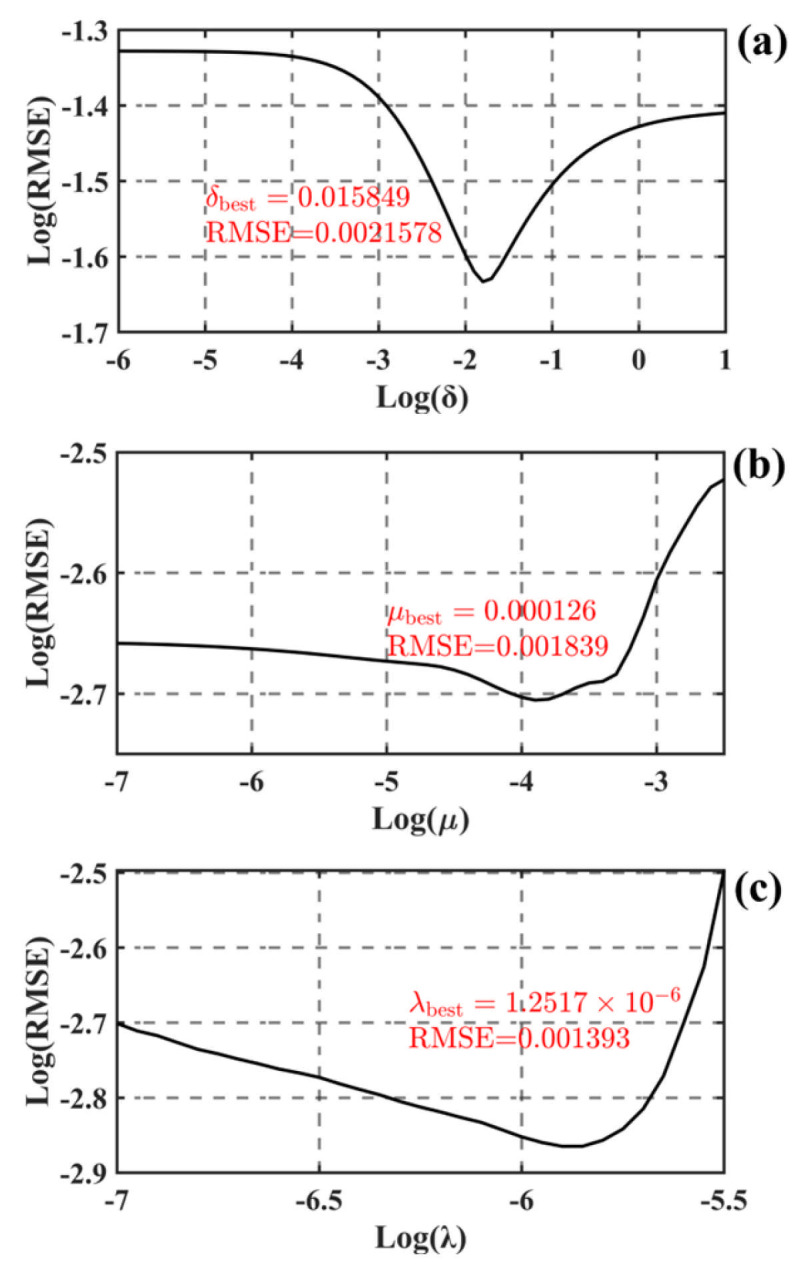
Results of hyperparameter testing for the noise-free model. RMSE as a function of hyperparameters. (**a**) RMSE versus *δ* with *μ* = 0, *λ* = 0; (**b**) RMSE versus *μ* with *δ* = 0.0158, *λ* = 0; (**c**) RMSE versus *λ* with *δ* = 0.0158, *μ* = 0.000126.

The investigation commenced by deactivating the HOLp and OGS regularization terms (i.e., setting their respective hyperparameters *μ* and *λ* to zero). The hyperparameter *δ* for the low-frequency background model term was then varied from 10^−7^ to 10, with an incremental step size of 0.1 on a logarithmic scale. The inversion was conducted under noise-free conditions. The RMSE between the inversion result and the true model was computed using Equation (29) for each *δ* value. The resulting RMSE curve is shown in Figure 5a. The analysis reveals that the RMSE reaches its minimum within the *δ* interval [10^−2.2^, 10^−1.5^], indicating superior agreement between the inversion result and the true model. The global minimum RMSE was achieved at *δ* = 0.0158. Subsequently, with *δ* fixed at this optimal value of 0.0158, the hyperparameter *μ* for the HOLP regularization term was examined. The value of *μ* was varied from 10^−8^ to 10^−1^ with a step size of 0.05. The corresponding RMSE curve is presented in Figure 5b. The results demonstrate that the minimum RMSE is attained when *μ* lies within the range [10^−4.5^, 10^−3.5^], with the most accurate result (RMSE = 0.0018) occurring at *μ* = 0.000126. Finally, with both *δ* and *μ* fixed at their optimal values (*δ* = 0.0158, *μ* = 0.000126), the hyperparameter *λ* for the OGS regularization term was optimized. The parameter *λ* was varied across the range [10^−8^ 10^−1^]. As illustrated in the RMSE curve in Figure 5c, the minimum error is achieved when *λ* falls within [10^−6^, 10^−5.6^], with the absolute minimum RMSE of 0.0014 observed at *λ* = 1.25 × 10^−6^.

**Figure 6:**
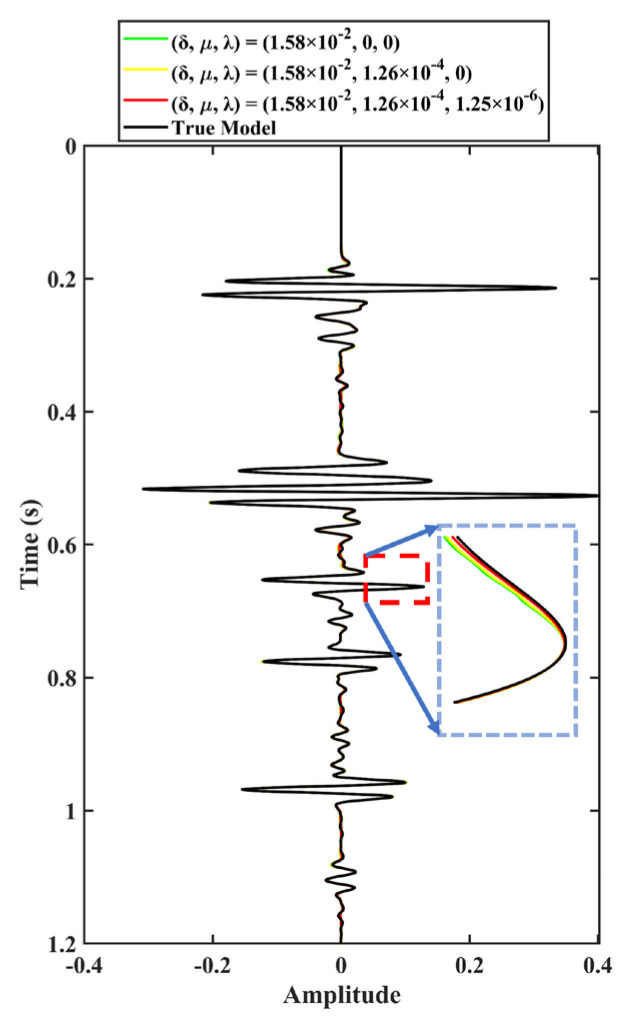
Single-trace inversion results under different hyperparameter configurations for the noise-free model. Green curve: *δ* = 0.0158, *μ* = 0, *λ* = 0; yellow curve: *δ* = 0.0158, *μ* = 0.0001, *λ* = 0; red curve: *δ* = 0.0158, *μ* = 0.000126, *λ* = 1.25 × 10^−6^; black curve: true model. The light blue dashed box shows a locally magnified view of the area within the red dashed box.

Figure 6 displays the single-trace inversion results corresponding to the sequential fixation of these hyperparameters. The green, yellow, and red curves in Figure 6 represent the inversion results associated with the minimum RMSE values from Figure 5a, Figure 5b and Figure 5c, respectively, while the black curve denotes the true model. The light blue inset provides a magnified view of the area highlighted by the red rectangle. It is evident that, under noise-free conditions, the inversion result converges progressively closer to the true model as each hyperparameter is optimally determined.

**Figure 7:**
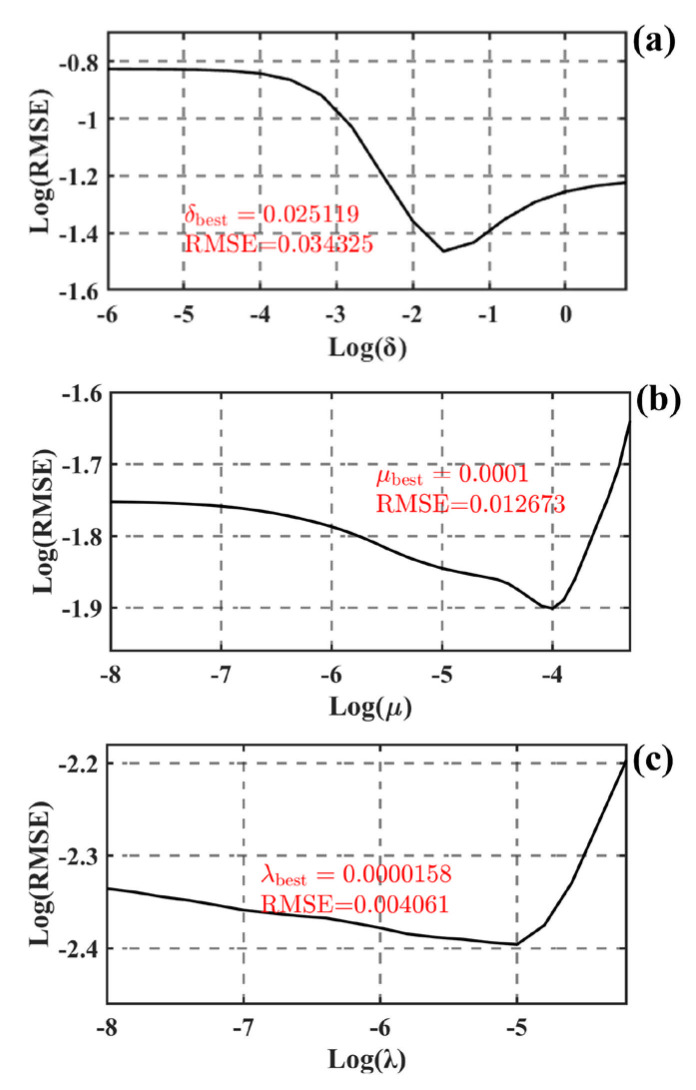
Results of hyperparameter testing when SNR = 2. RMSE as a function of hyperparameters. (**a**) RMSE versus *δ* with *μ* = 0, *λ* = 0; (**b**) RMSE versus *μ* with *δ* = 0.0251, *λ* = 0; (**c**) RMSE versus *λ* with *δ* = 0.0251, *μ* = 0.0001.

**Figure 8:**
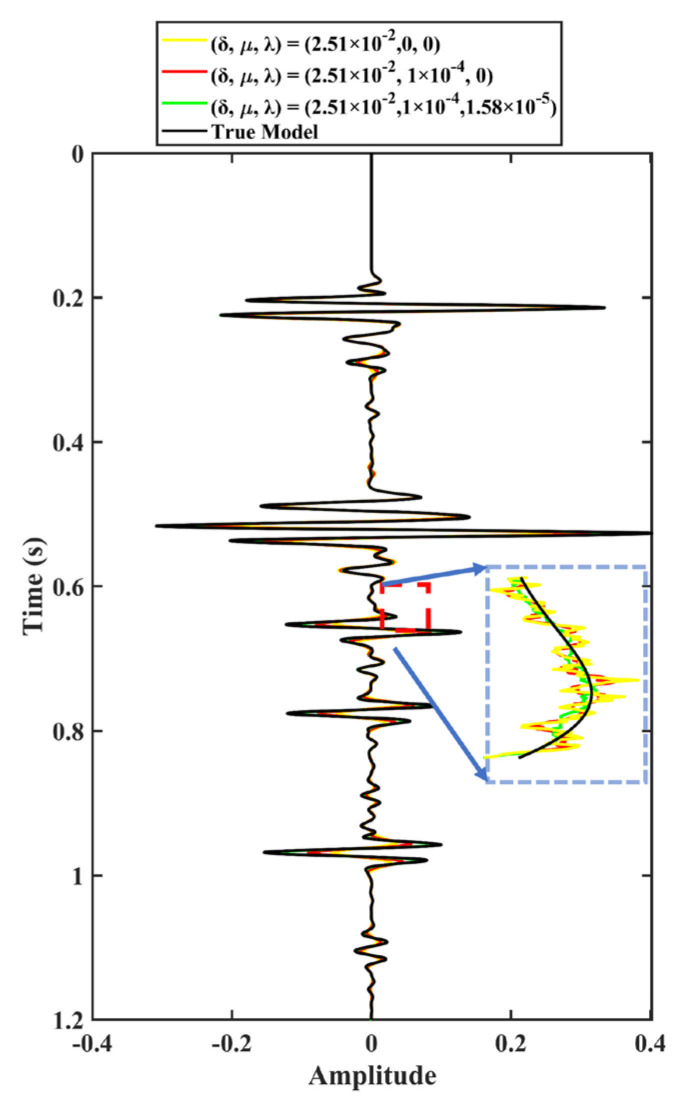
Single-trace inversion results under different hyperparameter configurations when SNR = 2. Yellow curve: *δ* = 0.0251, *μ* = 0, *λ* = 0; red curve: *δ* = 0.0251, *μ* = 0.0001, *λ* = 0; green curve: *δ* = 0.0251, *μ* = 0.0001, *λ* = 1.58 × 10^−5^; black curve: true model. The light blue dashed box shows a locally magnified view of the area within the red dashed box.

To further evaluate the robustness of the regularization parameter selection under low SNR conditions, the same procedure was repeated with noise added to achieve an SNR of 2. The resulting RMSE curves and the single-trace inversion comparison for this noisy scenario are presented in Figure 7 and Figure 8, respectively. The analysis indicates that satisfactory inversion performance under these noisy conditions is achieved when *δ* is within [10^−2^, 10^−1^], *μ* is within [10^−4.4^, 10^−3.8^], and *λ* is within [10^−5.4^, 10^−4.8^]. As shown in the light blue dashed box inset of Figure 8, using only the low-frequency background model constraint yields suboptimal results. However, the introduction of the HOLp constraint, followed by the combined application of HOLp and OGS constraints, not only enhances the noise immunity of the inversion but also improves the resolution of subtle wavefield details.

**Figure 9:**
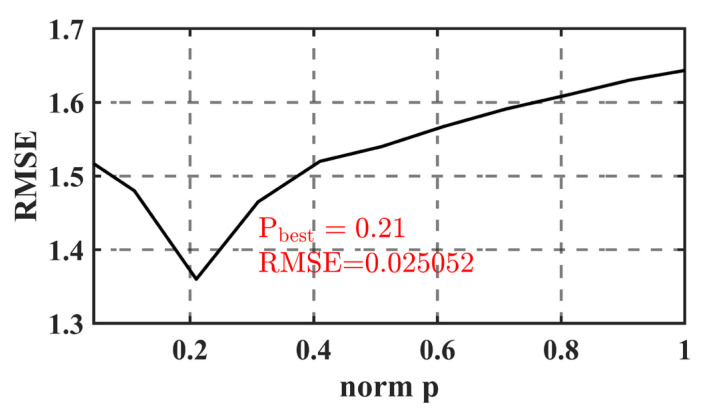
RMSE versus *p* for the noise-free model.

**Figure 10:**
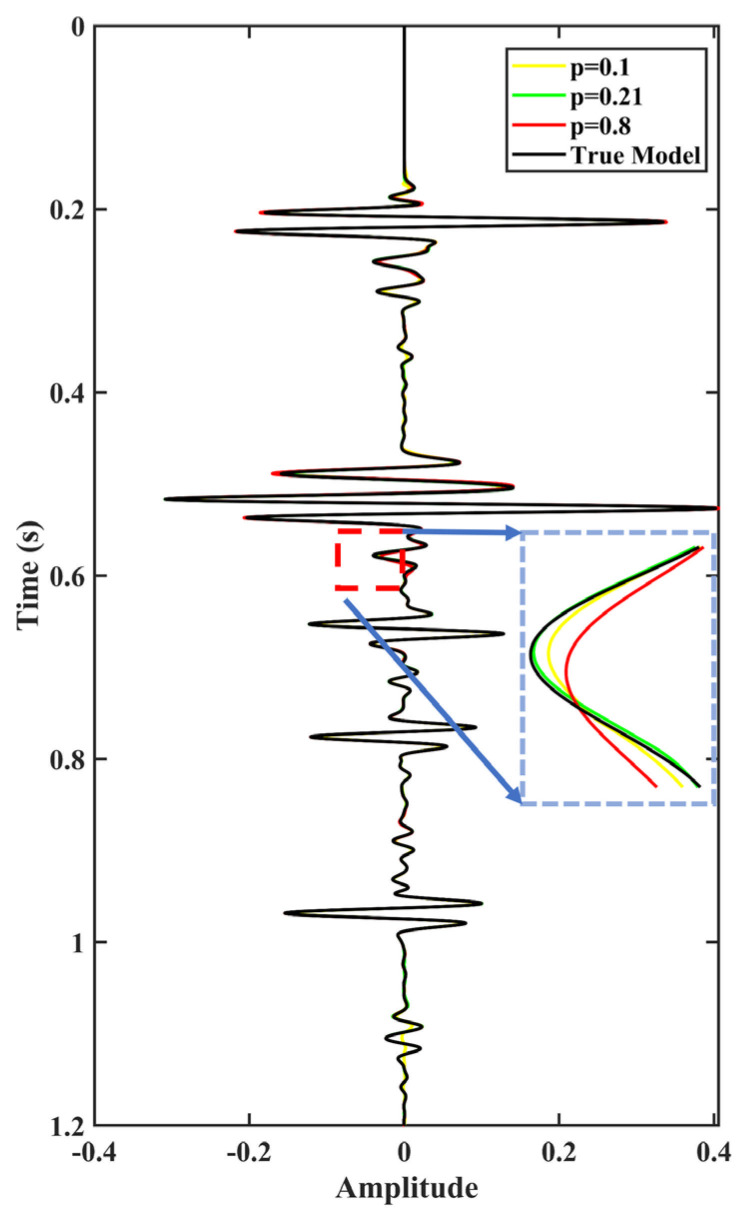
Single-trace comparison of inversion results under different parameter *p* in noise-free conditions. Yellow curve: *p* = 0.1; green curve: *p* = 0.21; red curve: *p* = 0.8; black curve: true model. The light blue dashed box shows a locally magnified view of the area within the red dashed box.

Furthermore, additional ablation studies were conducted to independently investigate the influence of the parameter *p* in the HOLP regularization and the value of the sparse groups *J* on the inversion results. Initially, the three primary hyperparameters were fixed, and *λ* was set to zero to examine how the RMSE curve changes as the parameter *p* varies within the interval [0, 1]. As shown in Figure 9, the RMSE of the inversion result first decreases and then increases as the parameter *p* increases with a step size of 0.1. The minimum RMSE is achieved at *p* = 0.21, indicating the optimal inversion performance for this configuration. Figure 10 presents single-trace inversion results for different parameter *p*, with a magnified view of certain details. It can be observed that the green curve, representing *p* = 0.21, exhibits the best agreement with the true model (black curve).

**Figure 11:**
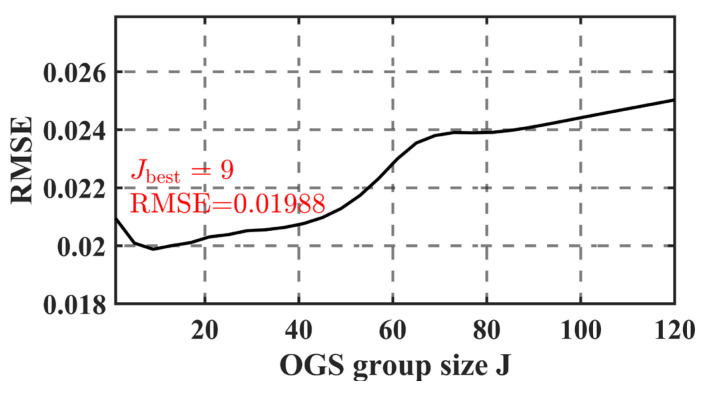
RMSE versus *J* for the noise-free model.

**Figure 12:**
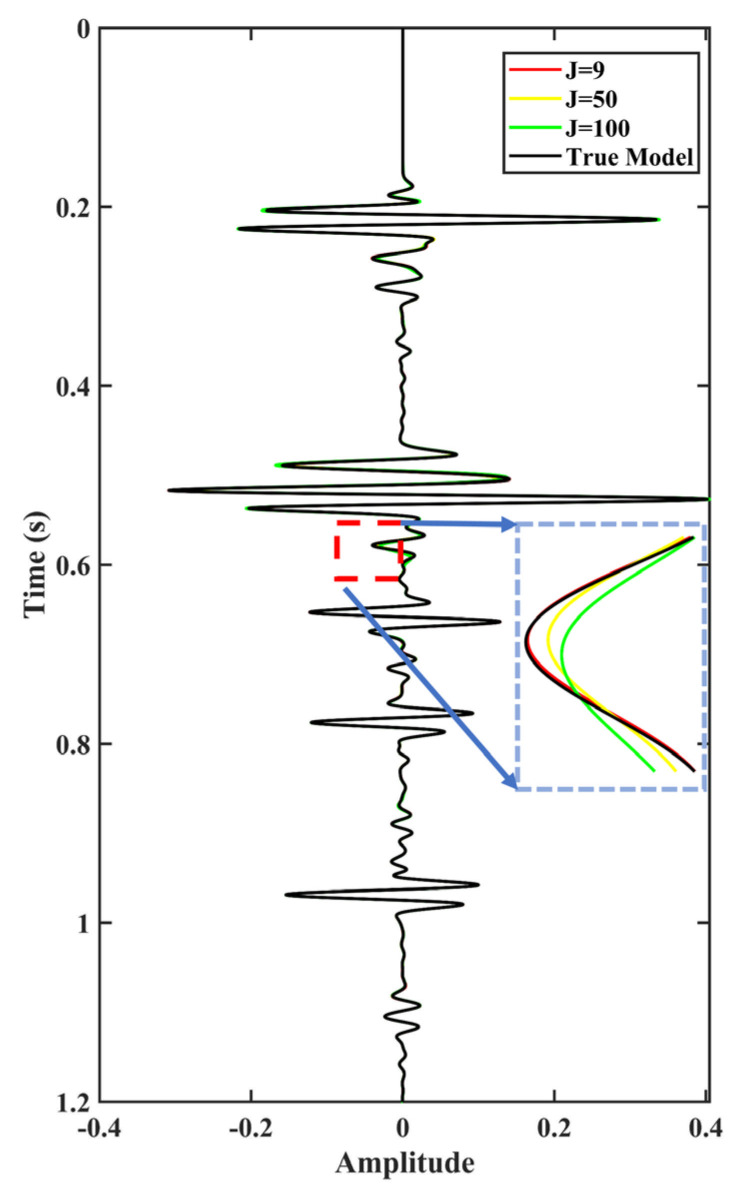
Single-trace comparison of inversion results under different *J*-values in noise-free conditions. Red curve: *J* = 9; yellow curve: *J* = 50; green curve: *J* = 100; black curve: true model. The light blue dashed box shows a locally magnified view of the area within the red dashed box.

Subsequently, *μ* was set to zero to investigate the impact of the number of sparse groups *J*. The RMSE variation was analyzed as *J* ranges from 0 to 120 with a step size of 5. The results, displayed in Figure 11, show that the RMSE reaches its minimum when *J* = 9, signifying the best inversion result. Figure 12 illustrates the single-trace inversion results for different *J*-values, with an enlarged section for detailed comparison. The red curve, corresponding to *J* = 9, aligns most closely with the true model (black curve). When the value of *J* exceeds 9, the RMSE gradually increases, with a noticeable abrupt change occurring near *J* = 40. Therefore, for the dataset under investigation, the optimal range for *J* is suggested to be between 5 and 20.

**Figure 13:**
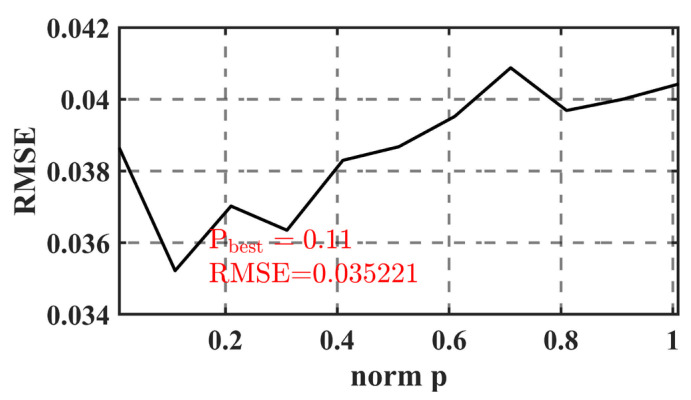
RMSE versus *p* when SNR = 2.

**Figure 14:**
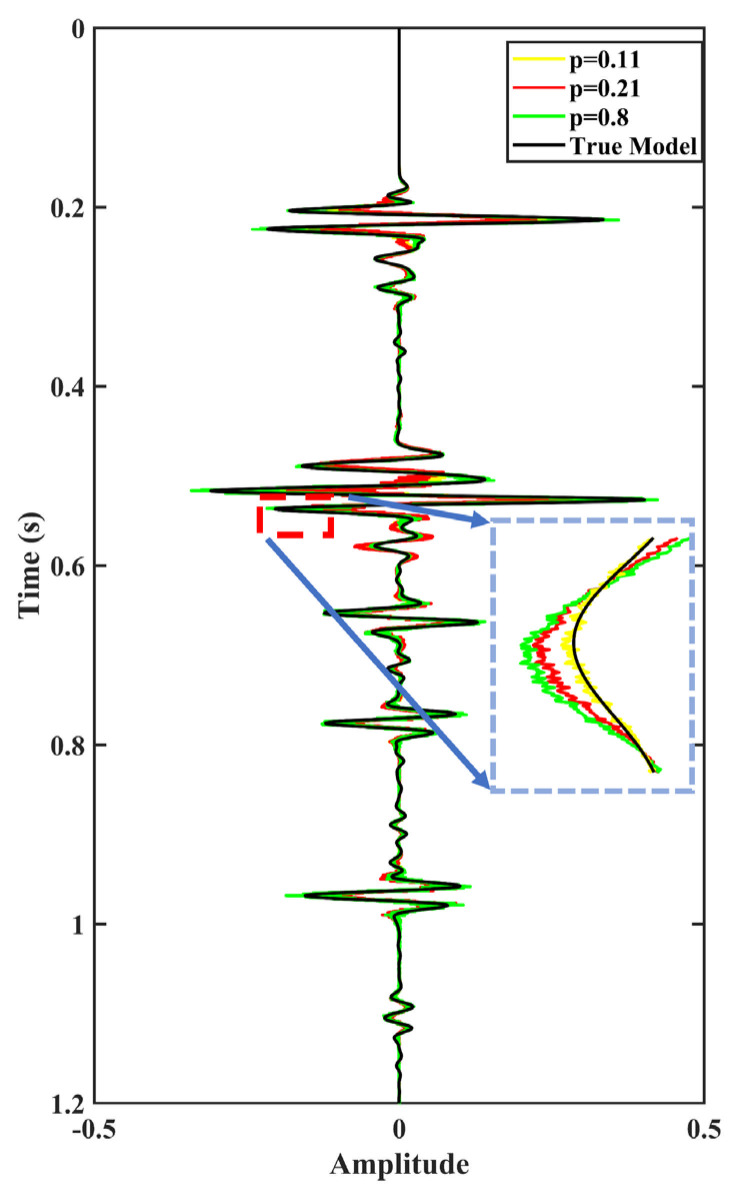
Single-trace comparison of inversion results under different parameter *p* when SNR = 2. Yellow curve: *p* = 0.11; red curve: *p* = 0.21; green curve: *p* = 0.8; black curve: true model. The light blue dashed box shows a locally magnified view of the area within the red dashed box.

The experimental methodology and workflow described previously were consistently applied to investigate the impact of the parameter *p* on inversion results under SNR = 2. The corresponding results are presented in Figure 13 and Figure 14. Analysis of the RMSE variation with respect to *p* reveals that smaller values of *p* generally correspond to lower RMSE, indicating enhanced anti-noise capability in the inversion results under these noisy conditions. As the value of *p* increases, the RMSE exhibits an overall increasing trend characterized by a segmented or zigzag pattern. A local minimum RMSE is observed at *p* = 0.11.

**Figure 15:**
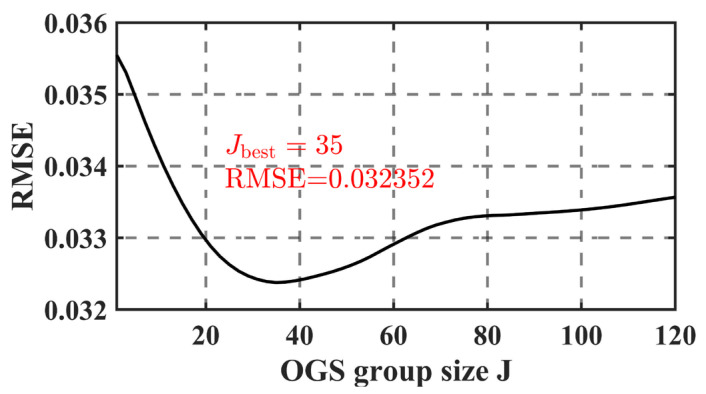
RMSE versus *J* when SNR = 2.

**Figure 16:**
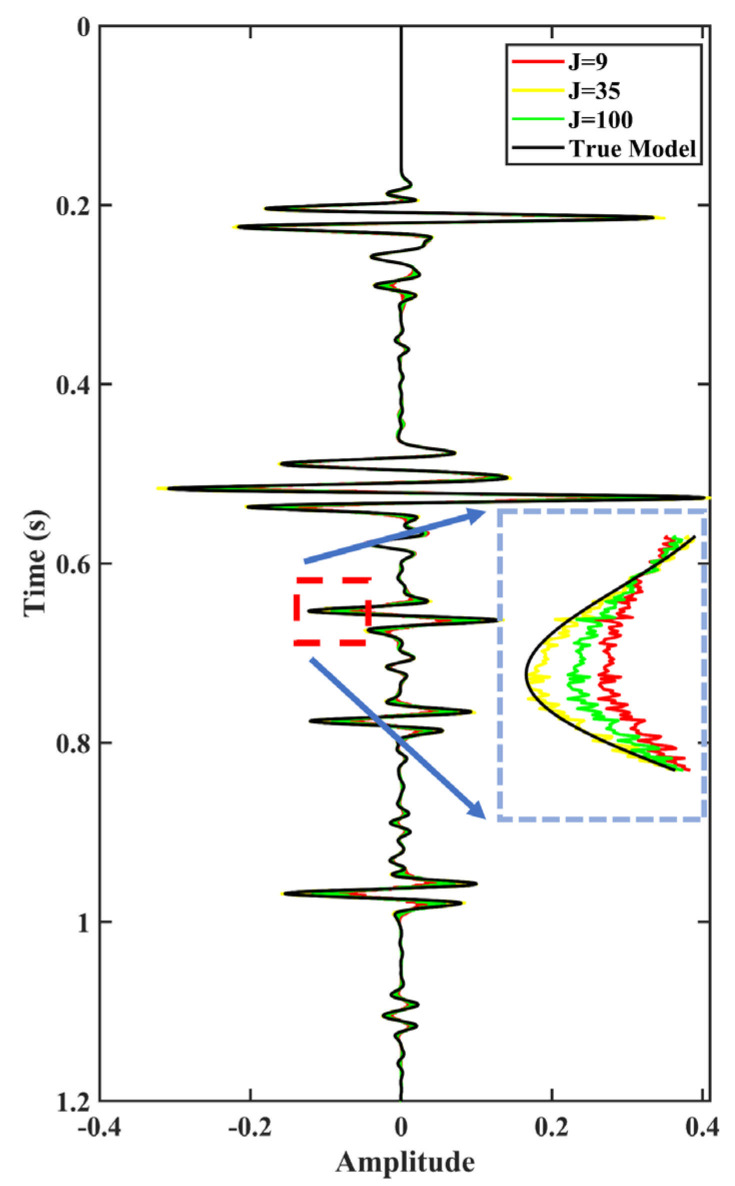
Single-trace comparison of inversion results under different *J*-values when SNR = 2. Red curve: *J* = 9; yellow curve: *J* = 50; green curve: *J* = 100; black curve: true model. The light blue dashed box shows a locally magnified view of the area within the red dashed box.

The influence of the parameter *J* on the inversion results under the same low SNR condition is shown in Figure 15. The RMSE reaches a local minimum when *J* = 35. Figure 16 provides a comparison of single-trace inversion results for different values of *J*. The enlarged view within this figure focuses on key details and shows good consistency with the trends displayed in Figure 15.

### 3.2. Synthetic Examples Tests

**Figure 17:**
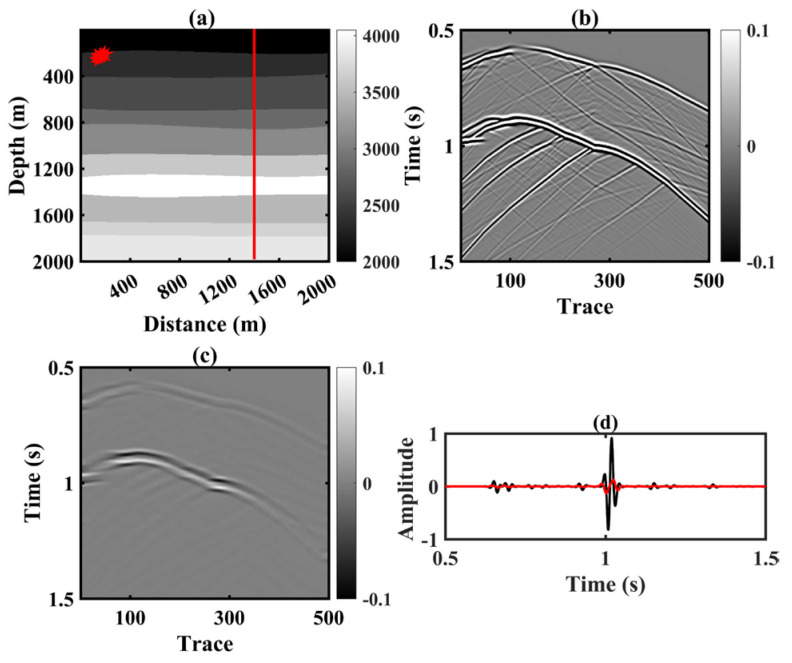
(**a**) Schematic of the stratigraphic model and acquisition geometry. The red explosion symbol denotes the source, whereas the red vertical line indicates the fiber deployment location; (**b**) synthetic seismic records; (**c**) low-frequency background model; and (**d**) data and initial model at trace 300 (black line: true value; red line: initial model).

To evaluate the efficacy of the proposed method, synthetic seismic records were simulated on a multilayered model using a first-order velocity-stress elastic wave equation with a 30 Hz Ricker wavelet. The model schematic and acquisition geometry are illustrated in Figure 17a, while Figure 17b displays the resulting synthetic records. Figure 17c shows the low-frequency background model used for inversion. In Figure 17d, the black and red lines denote the seismic data and the initial model at trace 300, respectively.

**Figure 18:**
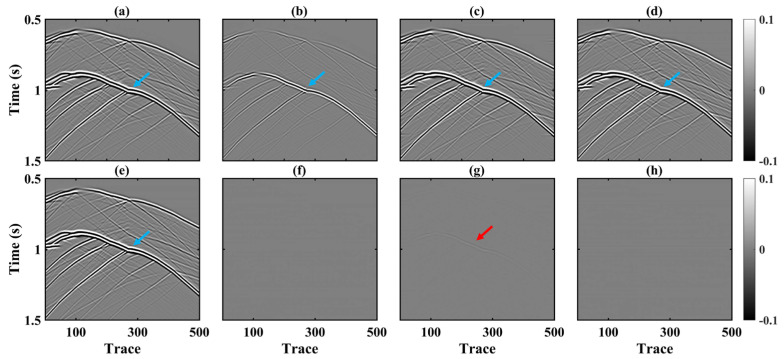
Synthetic example under noise-free conditions: (**a**) synthetic geophone-equivalent particle-velocity data; (**b**) synthetic DAS strain-rate data; (**c**) inversion result using the proposed HOLp–OGS method; (**d**) inversion result using *L*_2_ regularization; (**e**) inversion result using *L*_1_ regularization; and (**f**–**h**) residuals between the inversion results and the reference geophone-equivalent particle-velocity data for the HOLp–OGS, *L*_2_, and *L*_1_ methods, respectively.

**Figure 19:**
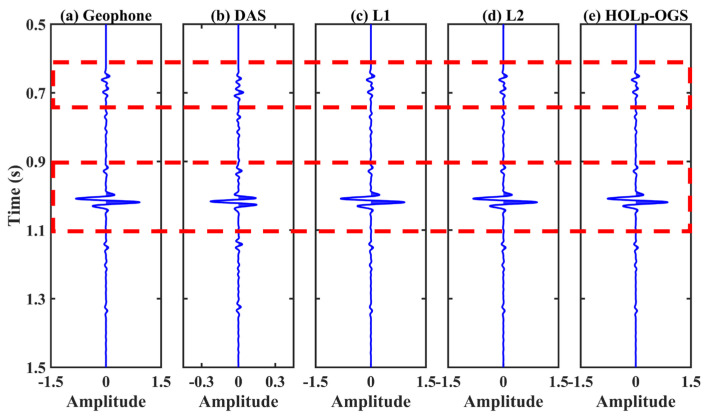
Comparison of strain-rate-to-velocity inversion results for the 300th trace under noise-free conditions: (**a**) synthetic geophone-equivalent velocity trace; (**b**) synthetic DAS strain-rate trace; (**c**) *L*_1_-regularized inversion result; (**d**) *L*_2_-regularized inversion result; and (**e**) HOLp–OGS-regularized inversion result. The red dashed boxes highlight several representative waveform comparisons.

A noise-free scenario was first considered to assess baseline inversion performance. Figure 18a depicts the synthetic particle velocity records (identical to Figure 17b). Figure 18b displays the DAS data derived via Equation (1), which exhibits a distinct polarity reversal relative to the particle velocity. The inversion results using HOLp–OGS, *L*_2_, and *L*_1_ regularizations are presented in Figure 18c–e, respectively. Table 1 details the quantitative metrics: the corresponding CC values are 99.92, 98.96, and 99.14, with RMSE values of 0.0025, 0.0036, and 0.0027, respectively. As indicated by the blue arrows, all methods successfully restore the correct waveform polarity. Figure 18f–h show the residuals between the inversion results and true models. Although all three methods yield accurate reconstructions under noise-free conditions, the HOLp–OGS regularization achieves superior fidelity with minimal residuals, whereas the *L*_2_ approach shows a slight mismatch (red arrow, Figure 18g). Figure 19 presents a trace-by-trace comparison at trace 300, further confirming that all methods accurately reproduce the true waveform, consistent with the 2D results in Figure 18.

**Figure 20:**
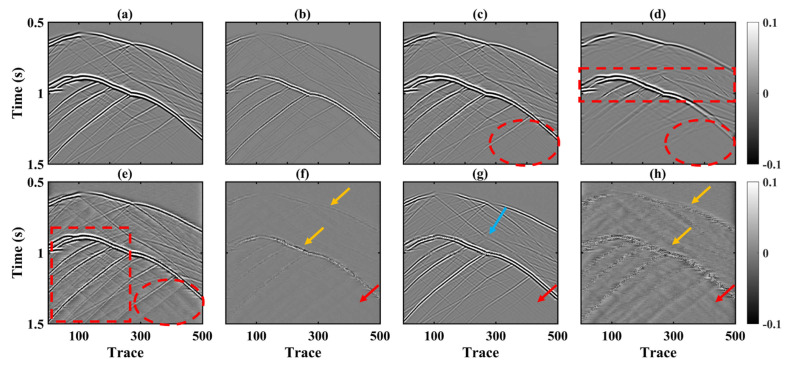
Synthetic example with noisy DAS data at an SNR of 2: (**a**) synthetic geophone-equivalent particle-velocity data; (**b**) noisy synthetic DAS strain-rate data; (**c**) inversion result using the proposed HOLp–OGS method; (**d**) inversion result using *L*_2_ regularization; (**e**) inversion result using *L*_1_ regularization; and (**f**–**h**) residuals between the inversion results and the reference geophone-equivalent particle-velocity data for the HOLp–OGS, *L*_2_, and *L*_1_ methods, respectively.

**Figure 21:**
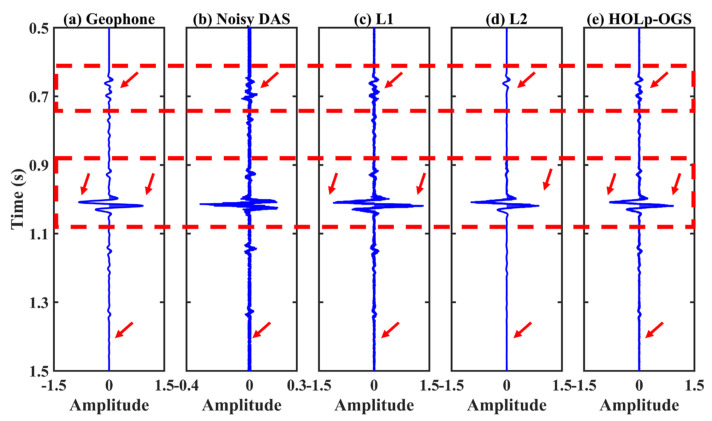
Comparison of strain-rate-to-velocity inversion results for the 300th trace at SNR = 2: (**a**) synthetic geophone-equivalent velocity trace; (**b**) noisy synthetic DAS strain-rate trace; (**c**) *L*_1_-regularized inversion result; (**d**) *L*_2_-regularized inversion result; and (**e**) HOLp–OGS-regularized inversion result. The red dashed boxes and arrows highlight several representative waveform comparisons.

To further evaluate the robustness of the proposed method, synthetic DAS data were corrupted with additive Gaussian noise corresponding to an SNR of 2. The results are presented in Figure 20 and Figure 21. The corresponding RMSE values are 0.0177, 0.0479, and 0.0354 (Table 1), with SNR values of 11.45, 2.95, and 8.07 dB, respectively. Under strong direct arrivals and severe noise, the *L*_2_-norm inversion fails to recover weak wavefield features, indicating limited robustness in low-SNR conditions (Figure 20d,g). The *L*_1_ result exhibits prominent noise artifacts and spurious events (red dashed square, Figure 20e), with residuals showing substantial mismatch (Figure 20h). In contrast, the proposed HOLp–OGS method better preserves weak signals and suppresses noise more effectively than the *L*_1_ approach, as indicated by the red dashed ellipses in Figure 20c,e. By comparison, the HOLp–OGS residuals contain only minor deviations and reduced background noise (Figure 20f).

Figure 21 presents single-trace comparisons. Figure 21b displays the noisy single-trace DAS data with SNR = 2 compared to the noise-free geophone data (Figure 21a). Due to significant noise interference in the near-zero amplitude regions of the DAS data, the noisy DAS trace exhibits apparent linewidth broadening and severe waveform distortion in the high-amplitude zones when visualized using identical plotting parameters. The *L*_1_ method recovers primary events but introduces noise and overestimates amplitude (0.9–1.1 s). The *L*_2_-norm inversion yields the smoothest result but fails to recover weak seismic events (0.6–0.73 s). HOLp–OGS yields the best result, recovering both strong and subtle features with minimal noise.

### 3.3. Field Data Test

The proposed method was further evaluated using field data from the Groß Schönebeck geothermal site in Germany [50]. The field dataset includes DAS records, borehole geophone check-shot records, and survey-geometry information from the GrSk3 and GrSk4 wells. In this study, we used the GrSk4 DAS data and the corresponding borehole geophone records. The geophone data were acquired with a Schlumberger Versatile Seismic Imager (VSI) tool, which is a three-component borehole geophone with acceleration response. Four check-shot records were acquired at measured depths of 1200, 2400, 3600, and 4207 m. For the single-trace comparison, we used the vertical component of the VSI data, which is parallel to the tool and borehole axis.

The original geophone acceleration records have a 5 s recording length and a 2 ms sampling interval. During preprocessing, a 4 s time window was extracted to match the DAS records used in this study. The DAS data were acquired in the same well with a Schlumberger hDVS system. The original hDVS output is strain data, which was differentiated in time to obtain strain-rate data. The strain-rate data were then used as the input to the proposed DAS-to-geophone conversion method. The method reconstructs geophone-equivalent particle-velocity data, which were further differentiated in time to obtain DAS-derived acceleration traces for comparison with the VSI acceleration data.

The DAS records used here have a 40 m gauge length, 5 m channel spacing, 2 ms sampling interval, and 4 s recording length. For each geophone depth, the nearest DAS channel was selected; thus, the nominal depth mismatch is no larger than half the DAS channel spacing. Because geophone records are available only at the four check-shot depths, CC and RMSE were computed only at these co-located positions rather than over the entire DAS section. When an overall metric is reported, it represents the average over the four available geophone depths. Before calculating CC and RMSE, the DAS-derived acceleration traces and the corresponding VSI acceleration traces were aligned and amplitude-normalized. No additional method-specific denoising was applied to the geophone reference traces. The full DAS section was evaluated qualitatively in terms of event continuity, noise attenuation, and waveform consistency.

**Figure 22:**
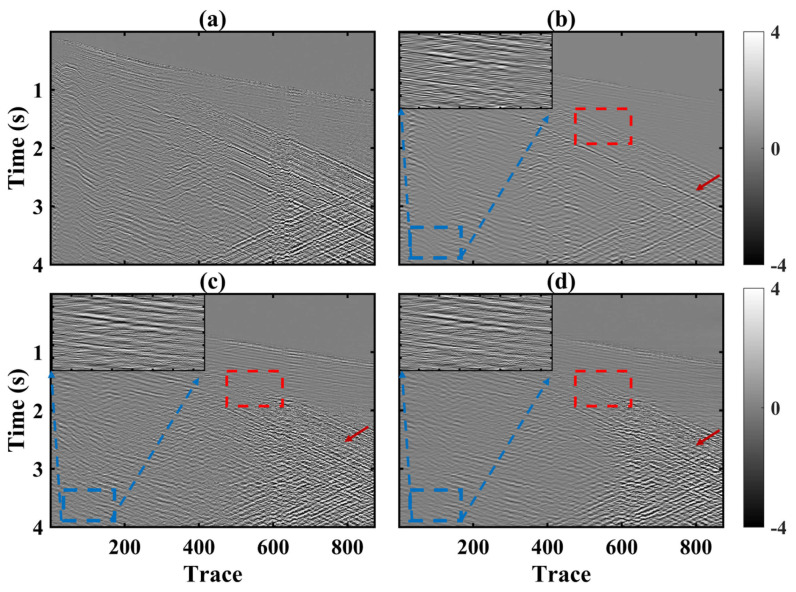
Comparison of field-data inversion results obtained with different regularization methods: (**a**) input field DAS data; (**b**) inversion result using the proposed HOLp–OGS method; (**c**) inversion result using *L*_2_ regularization; (**d**) inversion result using *L*_1_ regularization. The blue dashed boxes mark the regions enlarged in the upper-left insets, whereas the red dashed boxes indicate the regions further enlarged in the 3 × 1 zoomed panels. The dark red arrows highlight representative weak or structurally complex reflection events.

Figure 22 compares the inverse-reconstruction results obtained using HOLp–OGS, *L*_2_, and *L*_1_ regularization, and the corresponding CC and RMSE values are reported in Table 2. Relative to the tested *L*_1_- and *L*_2_-regularized results, the HOLp–OGS result contains fewer visible incoherent fluctuations and shows clearer weak events in the enlarged regions. These qualitative observations are consistent with its higher CC and lower RMSE values at the four evaluated geophone depths.

**Figure 23:**
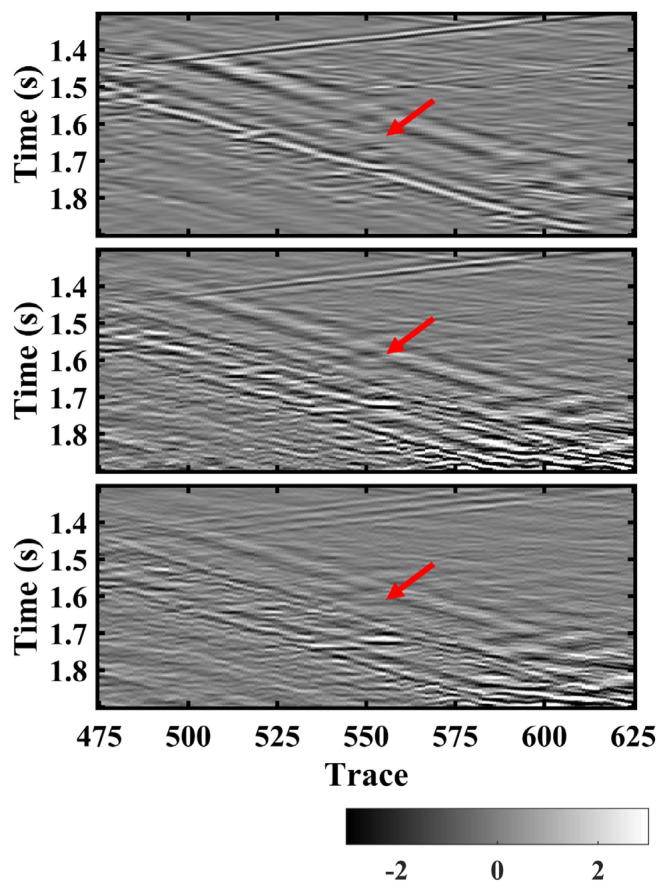
Zoomed comparison of the inversion results within the red dashed boxes in Figure 22: from top to bottom, the panels correspond to the results obtained with HOLp–OGS, *L*_2_-norm, and *L*_1_-norm regularization, respectively. A reduced amplitude clipping range is applied to enhance the visibility of weak reflection events. The red arrows highlight the comparison between the direct-wave arrivals.

Figure 23 presents enlarged views of the regions marked by the red dashed boxes in Figure 22. Under the same display scale, the HOLp–OGS result shows clearer first-arrival boundaries and more continuous weak events than the tested single-regularization results. The dark red arrows in Figure 22 highlight representative regions in which this improvement is visible.

To further evaluate the proposed method on field data, we compare it with a conventional f-k domain rescaling method for converting DAS strain-rate records to particle velocity. This method is an established DAS-to-geophone conversion approach rather than a simple f-k denoising filter. Similar f-k rescaling strategies have been used in previous DAS studies to relate DAS strain or strain-rate measurements to conventional ground-motion quantities or geophone records [17,19].

**Figure 24:**
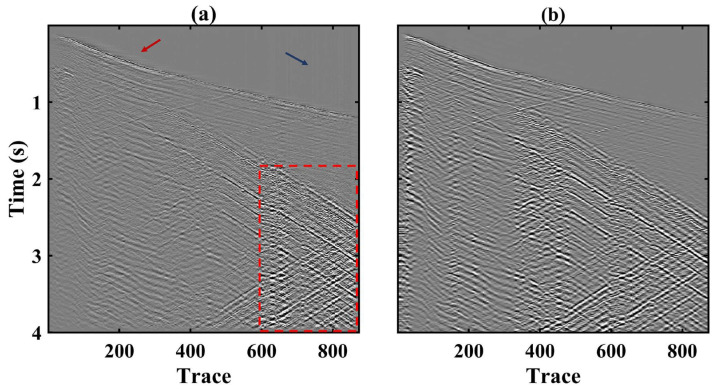
Comparison of field-data conversion results obtained with different methods: (**a**) conversion result using f-k rescaling method; and (**b**) HOLp–OGS-regularized inversion result. For a fair comparison, all results are amplitude-normalized.

Figure 24 compares the f-k rescaled result with the HOLp–OGS inverse-reconstruction result. The f-k method recovers the dominant dipping event and requires only 0.00175 s per trace on the same computational platform, demonstrating its substantial computational advantage. However, the f-k rescaled result retains visible spikes near the first arrivals and incoherent fluctuations in the noisy and structurally complex regions marked in Figure 24. In comparison, the HOLp–OGS result shows fewer visible fluctuations and more continuous weak events. These results indicate that, although the proposed method requires more computation than f-k rescaling, its improved noise attenuation, waveform fidelity, and weak-event preservation justify the additional computational cost. Moreover, its computation time remains comparable to that of the *L*_2_-regularized method and substantially lower than that of the *L_1_*-regularized method (Table 2), demonstrating a favorable balance between reconstruction quality and computational efficiency.

**Figure 25:**
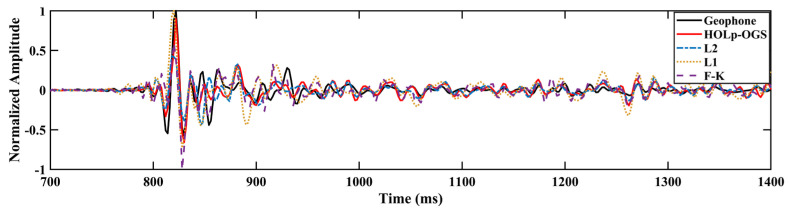
Comparison of normalized field-data conversion results obtained with HOLp–OGS, *L*_2_-norm, and *L*_1_-norm regularization and f-k rescaling method, along with the corresponding field geophone data.

**Figure 26:**
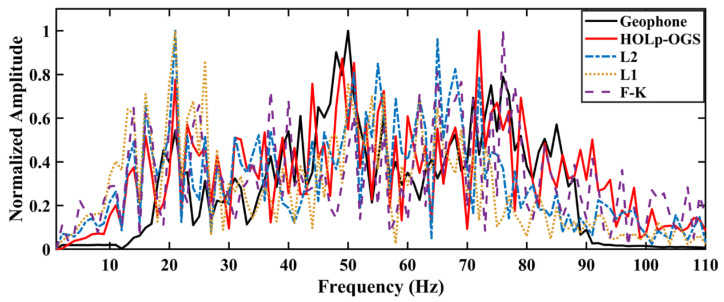
Single-trace comparison of normalized amplitude spectra for DAS-to-geophone conversion results obtained with different methods, together with the corresponding field geophone data.

The single-trace waveform comparison in Figure 25 and the corresponding amplitude-spectrum comparison in Figure 26, extracted at a depth of 2400 m, indicate that the HOLp–OGS inversion result provides the closest match to the co-located geophone measurement. After compensation for the gauge-length-induced filtering effect, the remaining discrepancies primarily reflect differences among the conversion methods. The improved agreement in both the time and frequency domains demonstrates the reliability of the proposed HOLp–OGS method.

## 4. Discussion

In the proposed framework, the HOLp penalty promotes a compact representation of the corresponding higher-order difference coefficients, thereby limiting noise-driven fluctuations in the reconstructed result. The OGS penalty jointly constrains overlapping groups of neighboring difference coefficients and helps reduce isolated artifacts while preserving locally continuous waveform variations. The two penalties therefore provide complementary constraints that balance noise attenuation and waveform preservation more effectively than the tested single-regularization methods. Nevertheless, the proposed method still has two main limitations. The first is related to computational efficiency. Although its computational cost is comparable to, and in some cases slightly lower than (Table 2), that of other advanced regularization-based schemes, processing large-volume DAS datasets remains challenging because of the dense spatial sampling and long recording duration of DAS acquisitions. Future work may explore hybrid physics-guided deep learning strategies that combine the proposed inversion framework with learned DAS-to-geophone conversion modules or network-based acceleration schemes. Such a framework could improve computational efficiency while alleviating the limited cross-survey generalization capability of purely data-driven methods, thereby maintaining physical consistency and inversion stability.

The second limitation concerns the incorporation of DAS angular sensitivity. Under the ideal plane-wave assumption, the DAS response to a P-wave is approximately proportional to $\cos^{2}\theta$, where θ denotes the angle between the fiber axis and the wave-propagation direction. In contrast, the response of a conventional single-component geophone is approximately proportional to cosθ [7,51]. As a result, DAS amplitudes decay more rapidly than geophone amplitudes with increasing incident angle. Similar to many conventional DAS-to-geophone conversion methods, the present study focuses on the conversion problem itself and does not explicitly account for angle-dependent amplitude attenuation. In existing workflows, DAS angular sensitivity is commonly handled as a separate correction or processing step. However, inaccurate correction may introduce amplitude distortions that are subsequently propagated into inversion results. Future work will investigate the incorporation of DAS angular sensitivity into the proposed forward-modeling and inversion framework, thereby reducing the reliance on separate amplitude-correction procedures.

## 5. Conclusions

This study investigates the DAS-to-geophone conversion problem in distributed acoustic sensing data, which is essential for integrating DAS measurements into conventional seismic processing workflows. DAS-to-geophone conversion requires compensation for the finite-gauge-length measurement response, while noise in field DAS records makes stable waveform recovery challenging. We therefore formulate the conversion as a regularized inverse-reconstruction problem.

To improve the robustness of inversion-based DAS-to-geophone conversion, we propose a framework that integrates HOLp and OGS regularizations within an efficient ADMM optimization scheme. The finite-gauge-length response is explicitly incorporated into the forward operator to establish the physical relationship between DAS observations and particle velocity. Field data experiments demonstrate that the proposed approach provides better performance than the conventional f-k filtering method and the *L*_1_- and *L*_2_-norm regularized inversion methods, achieving improved noise attenuation while preserving waveform continuity and amplitude fidelity.

Future work will focus on incorporating DAS angular sensitivity into the forward-modeling and inversion framework and on developing adaptive, computationally efficient optimization strategies to improve the scalability of the proposed method for large-volume field data.

## Figures and Tables

**Table 1:** RMSE, CC, and computation time per trace for model tests.

Method	Noise-Free Model			Noisy Model		
	RMSE	CC (%)	Computation Time (s)	RMSE	CC (%)	Computation Time (s)
*L*_1_	0.0027	99.14	0.1193	0.0354	85.28	0.1449
*L*_2_	0.0036	98.96	0.0526	0.0479	75.74	0.0683
HOLp–OGS	0.0025	99.92	0.0541	0.0177	95.86	0.0703

**Table 2:** RMSE, CC, and computation time per trace for field data tests among the inversion-based methods.

Method	Field Data		
	RMSE	CC (%)	Computation Time (s)
*L*_1_	0.1438	81.42	0.0938
*L*_2_	0. 2119	73.19	0.0404
HOLp–OGS	0.0826	92.13	0.0501

## Data Availability

The research data supporting this study are available upon request to interested researchers.

## Appendix A

### Solution Methods for Subproblem W

Solving the subproblem W in Equation (22) is equivalent to finding the optimal solution for the HOLp regularization problem, as expressed by:

(A1) $$W^{n+1}=\underset{W}{\mathrm{argmin}\;}\left(J_{W}\left(W\right)=\mu {\left\|W\right\|}_{p}^{p}-\alpha {\left(\alpha_{W}^{n}\right)}^{T}\left(W-G_{s}S^{n}\right)+\frac{\beta }{2}{\left\|W-G_{s}S^{n}\right\|}_{2}^{2}\right).$$

Applying the IRL_1_ algorithm to solve Equation (A1) yields Equation (A2):

(A2) $$K^{n+1}=G_{s}S^{n}+\alpha_{W}^{n}.$$

Equation (A1) can be rewritten by substituting Equation (A2) into it, resulting in:

(A3) $$W^{n+1}=\underset{W}{\mathrm{argmin}\;}\frac{\beta }{2}{\left\|W-K^{n}\right\|}_{2}^{2}+\mu {\left\|W\right\|}_{p}^{p}.$$

For each iteration, the approximate solution is computed via Equation (A4):

(A4) $$W^{n+1}=\underset{W}{\mathrm{argmin}}\frac{\beta }{2}∥W-K^{n}{∥}_{2}^{2}+\sum_{i=1}^{n}\mu p{\left(|W_{i}^{n}|+\xi \right)}^{p-1}|W_{i}|,$$

where ξ is a small constant introduced to ensure numerical stability by avoiding division by zero. In this study, we set ξ = 10^−7^. Furthermore, set:

(A5) $$t_{i}^{n+1}=\mu p{\left(|W_{i}^{n}|+\xi \right)}^{p-1}.$$

By substituting Equation (A5) into Equation (A4), we obtain:

(A6) $$W^{n+1}=\underset{W}{\mathrm{argmin}}\frac{\beta }{2}∥W-K^{n}{∥}_{2}^{2}+\sum_{i=1}^{n}t_{i}^{n+1}|W_{i}|,$$

We solve Equation (A6) using the iterative soft-thresholding algorithm, yielding the following equation:

(A7) $$W^{n+1}=\mathrm{shrink}\;\left(K^{n},\frac{\mu t^{n+1}}{\beta }\right)=\max\left\{K^{n}-\frac{\mu t^{n+1}}{\beta },0\right\}\cdot \mathrm{sign}\left(K^{n}\right).$$

where $\mathrm{sign}\left(K^{n}\right)$ denotes sign function.

## Appendix B

### Solution Methods for Subproblem R

The solution to subproblem **R** in Equation (22) is given by Equation (A8):

(A8) $$R^{n+1}=\underset{R}{\mathrm{argmin}\;}\left(J_{R}\left(R\right)=\lambda \varphi (R)-\beta {\left(\alpha_{R}^{n}\right)}^{T}\left(R-D_{s}S^{n}\right)+\frac{\beta }{2}{\left\|R-D_{s}S^{n}\right\|}_{2}^{2}\right).$$

To solve Equation (A8), we employ the optimization steps formulated in Equation (A9):

(A9) $$R^{n+1}=\underset{R}{\mathrm{argmin}\;}\left(J_{R}\left(R\right)=\frac{\beta }{2}{\left\|R-\left(D_{s}S^{n}+\alpha_{R}^{n}\right)\right\|}_{2}^{2}+\lambda \varphi (R)\right).$$

To solve Equation (A9), we employ the majorization–minimization (MM) algorithm. By combining a general inequality $a^{2}+b^{2}\geq 2ab$ with the definition of φ(R), we derive Equation (A10):

(A10) $$\Phi \left(R,U\right)=\frac{1}{2}\left({\left\|\sum_{t}\frac{R_{t,N}}{U_{t,N}}\right\|}_{2}^{2}+{\left\|U_{t,N}\right\|}_{2}\right),$$

where $\Phi \left(R,U\right)\geq \varphi (R)$, $\Phi \left(R,R\right)=\varphi (R)$ and ${\left\|U_{t,N}\right\|}_{2}\neq 0$. t denotes the number of elements in the set. The above expression can be further rewritten as:

(A11) $$\Phi \left(R,U\right)=\frac{1}{2}R^{T}\Lambda (U)R+C,$$

where C is independent of R and Λ(U) represents a diagonal matrix, defined as:

(A12) $${\left[\Lambda \left(u\right)\right]}_{t,t}={\left(\sum_{n=0}^{N-1}{\left|u\left(t+n\right)\right|}^{2}\right)}^{-1/2}$$

By substituting $\Phi \left(R,U\right)$ for φ(R) and inserting the result into Equation (A9), we obtain:

(A13) $$L_{R}(R,U)=\frac{\beta }{2}∥R-\left(DS^{n}+\alpha_{R}^{n}\right){∥}_{2}^{2}+\lambda \Phi \left(R,U\right)=\frac{1}{2}\left(\beta R-{\left(DS^{n}+\alpha_{R}^{n}\right)}_{2}^{2}+\lambda R^{T}\Lambda \left(U\right)R\right)+C,$$

where $L_{R}\left(R,U\right)\geq J_{R}\left(R\right)$ and $L_{R}\left(R,R\right)=J_{R}\left(R\right)$. According to the principles of the MM algorithm, minimizing $J_{R}\left(R\right)$ is equivalent to minimizing $L_{R}\left(R,R^{n}\right)$. Consequently, this leads to the following iterative update formula:

(A14) $$R^{n+1}=\underset{R}{\mathrm{argmin}}\left(L_{R}\left(R,R^{n}\right)\right)=\underset{R}{\mathrm{argmin}}\left(\beta ∥R-\left(DS^{n}+\alpha_{R}^{n}\right){∥}_{2}^{2}+\lambda R^{T}\Lambda \left(R^{n}\right)R\right),$$

The solution to Equation (A14) is given by:

(A15) $$R^{n+1}={\left(I+\frac{\beta }{\lambda }\Lambda {\left(R^{n}\right)}^{T}\Lambda \left(R^{n}\right)\right)}^{-1}\left(DS^{n}+\alpha_{R}^{n}\right).$$
